# Brain Derived Neurotrophic Factor and Cognitive Dysfunction in the Schizophrenia-Bipolar Spectrum: A Systematic Review and Meta-Analysis

**DOI:** 10.3389/fpsyt.2022.827322

**Published:** 2022-05-24

**Authors:** Zsófia B. Dombi, István Szendi, Philip W. J. Burnet

**Affiliations:** ^1^Department of Psychiatry, University of Oxford, Oxford, United Kingdom; ^2^Medical Division, Gedeon Richter Plc., Budapest, Hungary; ^3^Department of Psychiatry, Kiskunhalas Semmelweis Hospital, Kiskunhalas, Hungary; ^4^Department of Software Engineering, University of Szeged, Szeged, Hungary

**Keywords:** schizophrenia, schizoaffective disorder, bipolar disorder, BDNF, cognition, biomarker

## Abstract

**Background:**

Cognitive impairment is a core feature of disorders on the schizophrenia-bipolar spectrum, i.e., schizophrenia, bipolar disorder, and schizoaffective disorder. Brain-derived neurotrophic factor (BDNF) has been proposed to be a biomarker of cognitive impairment in these disorders as it plays a critical role in neuroplasticity and proposed to mediate some of the psychotropic effects of medication. However, despite numerous studies investigating the association between circulating BDNF and these disorders, no solid conclusions have been drawn regarding its involvement in cognitive impairment.

**Objectives:**

The current systematic review and meta-analysis aims to examine blood BDNF levels and cognitive dysfunction in patients on the schizophrenia-bipolar spectrum as well as to evaluate whether circulating BDNF measurements can act as a biomarker for cognitive dysfunction.

**Methods:**

Studies were identified by searching Embase and Medline databases for English language articles published in peer-reviewed journals between 2000 January and 2021 June according to the PRISMA guidelines. A total of 815 articles were identified of which 32 met the inclusion criteria for the systematic review – reporting on comparisons between blood BDNF levels and cognitive functions of schizophrenia or bipolar disorder patients versus healthy controls (no studies involving schizoaffective patients were specifically obtained for the time being). Twenty-four of these studies (19 with schizophrenia and 5 with bipolar disorder patients) were eligible to be included in the meta-analysis.

**Results:**

Our findings indicated that circulating BDNF levels were significantly reduced in patients experiencing an acute episode of schizophrenia or bipolar disorder compared to healthy controls. Cognitive function was also found to be significantly worse in patients, however, correlations between BDNF levels and cognitive impairment were not always detected. Interventions, especially pharmacotherapy seemed to improve certain aspects of cognition and increase circulating BDNF levels.

**Conclusion:**

Circulating BDNF alone does not seem to be a valid biomarker of cognitive dysfunction in patients with disorders on the schizophrenia-bipolar spectrum, owing to several confounding factors. Changes of the circulating levels of BDNF should be evaluated in a wider context of other stress-, immune-, and inflammatory-related factors.

## Introduction

Schizophrenia is a serious psychiatric disorder characterized by considerable distortions of thinking and perception driven by three core symptom domains; positive symptoms, negative symptoms, and cognitive dysfunction (1). Bipolar disorder is also a major psychiatric condition, but it is recognized by the alternation of mood episodes and behavioral activation (1). The prevalence of both disorders is around 1% of the general population (2, 3). An intermediate phenotype between schizophrenia and bipolar disorder is schizoaffective disorder, which is characterized by the concurrent occurrence of an equal admixture of both schizophrenic and major affective disorder symptoms cross-sectionally and/or longitudinally (1). Together, the three disorders can be referred to as schizophrenia-bipolar spectrum disorders.

Cognitive dysfunction, defined broadly as the inability to properly process information, has been well established to be a core feature of these disorders (4, 5). In bipolar disorder, cognitive impairment usually manifests in specific cognitive domains such as attention, verbal memory, or executive functioning with greater severity throughout the acute manic-depressive episodes compared to the euthymic states (6–8). In contrast, the same cognitive deficits in schizophrenia tend to be stable across time without considerable improvements between psychotic episodes (9, 10). Deficits in cognition have also been described in schizoaffective disorder and at a greater extent than in patients with bipolar disorder (11). Importantly, cognitive impairment has been proposed to be a crucial factor in achieving improved functioning and quality of life in these patient groups (12–14). However, since currently there are no effective treatments for cognitive impairment in the schizophrenia-bipolar spectrum, it remains a major unmet clinical need (15). Thus, investigating potential biomarkers of cognitive dysfunction is not only crucial for understanding the pathophysiology of disorders on the schizophrenia-bipolar spectrum, but also for the development of potential treatments and interventions (16, 17). One of the candidates for such biomarker is brain-derived neurotrophic factor (BDNF).

Brain-derived neurotrophic factor is a member of the nerve growth factor family, which functions include enhancing the growth and maintenance of various neuronal systems, ensuring neuronal plasticity, modulating neurotransmitter activity, and contributing to learning and memory throughout life (18–21). It facilitates neuronal plasticity *via* the stimulation of dendritic growth, the formation of synapses as well as neurogenesis in brain areas related to memory such as the hippocampus (21, 22). Activation of BDNF release from axons is influenced negatively by several factors including inflammation, stress as well as age (21). Since BDNF can be readily measured in blood, several studies have associated its peripheral concentrations with central functions and neuropathology. For instance, circulating BDNF levels have been associated with hippocampal volume and spatial memory in older adults, with lower levels of BDNF correlating with smaller volume of the hippocampus and worse performance on neurocognitive tests (23). Furthermore, in terms of stress, animal studies found that social isolation in mice resulted in decreased BDNF levels in several brain areas including the prefrontal cortex, hippocampus, and hypothalamus (24). In view of these results and that it can cross the blood–brain barrier, BDNF has gained considerable attention as a possible biomarker for neurocognitive processes in several psychiatric and neuropsychiatric disorders (25) such as Alzheimer’s disease (26), autism spectrum disorder (27), or disorders on the schizophrenia-bipolar spectrum (28).

Several studies measured the concentrations of BDNF in the blood and examined its relationship with cognitive symptoms in patients on the schizophrenia-bipolar spectrum compared to healthy controls in order to better understand the role of BDNF. The findings of such studies however were quite mixed; a meta-analysis by Ahmed et al. did not observe significant connection between cognitive impairment and BDNF levels in the blood based on five schizophrenia studies (29). Another systematic review and meta-analysis involving 21 studies with schizophrenia patients reported a positive correlation between cognitive impairment and reduced blood BDNF levels, especially in chronic samples (30). In the case of bipolar disorder, meta-analyses have consistently reported reduced BDNF levels in manic and depressive episodes compared to healthy controls (31, 32), but the connection between BDNF levels and cognition in these reviews were not examined.

The present systematic review and meta-analysis aims to update the existing literature regarding circulating BDNF levels and cognitive functioning in the schizophrenia-bipolar spectrum in comparison to healthy controls, in order to conclusively demonstrate whether blood BDNF can act as a biomarker of cognitive dysfunction.

## Methods

### Search Strategy

Studies for the systematic review were identified by searching Embase and Medline databases for English language articles published in peer-reviewed journals between 1 January 2000 and 1 June 2021 according to the PRISMA guidelines (33) with search terms “(schizo* OR bipolar) AND (BDNF* OR ‘Brain Derived Neurotrophic Factor’) AND (‘Neurocognit*’ OR Cognit*).” Searches by hand and *via* the reference section of published reports and previous review papers were also conducted in order to identify additional relevant studies.

### Inclusion and Exclusion Criteria

The inclusion criteria for studies were the following: (1) original research conducted with human subjects; (2) involved patients with diagnosis on the schizophrenia-bipolar spectrum; (3) included at least one cognitive assessment; (4) reported enzyme-linked immunosorbent assay (ELISA) measurement of BDNF levels in blood serum or plasma; (5) included healthy controls. Papers were excluded if they examined only genetic BDNF data or baseline blood BDNF levels were not adequately reported. Studies that did not report BDNF levels or cognitive scores for the total patient sample were excluded from the meta-analyses.

### Statistical Analyses

Means, standard deviations (SDs), and effect sizes were calculated in Microsoft Excel. The effect size was calculated for differences between baseline BDNF levels of schizophrenia patients and healthy controls or bipolar patients and healthy controls in ng/ml using mean, SD, and sample sizes. In case of cognitive scores, the effect size was based on the differences between baseline Repeatable Battery for the Assessment of Neuropsychological Status (RBANS) scores of schizophrenia patients and healthy controls using mean, SD, and sample sizes. As effect size measure, Hedge’s g was computed since the studies included in the meta-analyses had relatively small sample sizes and Hedge’s g is less biased in case when variance equality assumptions are not met.

The meta-analyses were performed using the “meta” package in R studio, with standardized mean difference used as effect size measurement. While *Z* statistic was calculated to determine the significance of the effect size, *Q* statistic was computed to provide an estimation of the degree of homogeneity of the effect sizes of the different studies. The degree of inconsistency was signalized with the *I*^2^ metric (*I*^2^ > 75% indicating large heterogeneity, >50% moderate heterogeneity, and <50% low heterogeneity). To present the effect sizes of individual studies, a forest plot was created.

Due to the heterogeneity of studies, separate analyses for schizophrenia and bipolar disorder patients were performed with three random-effects meta-analyses. The first analysis examined the difference in circulating BDNF levels between schizophrenia patients and healthy controls, the second examined the difference in circulating BDNF levels between bipolar disorder patients and healthy controls, and the third looked at the difference in cognitive functions measured by RBANS between schizophrenia patients and healthy controls. Data was scarce to conduct an analysis for the difference between bipolar disorder patients and healthy controls in terms of cognitive functions. Similarly, conducting a meta-analysis of correlation coefficients was impossible due to the scarcity and heterogeneity of data. In these cases, qualitative analyses were performed.

## Results

### Search Results

**FIGURE 1:**
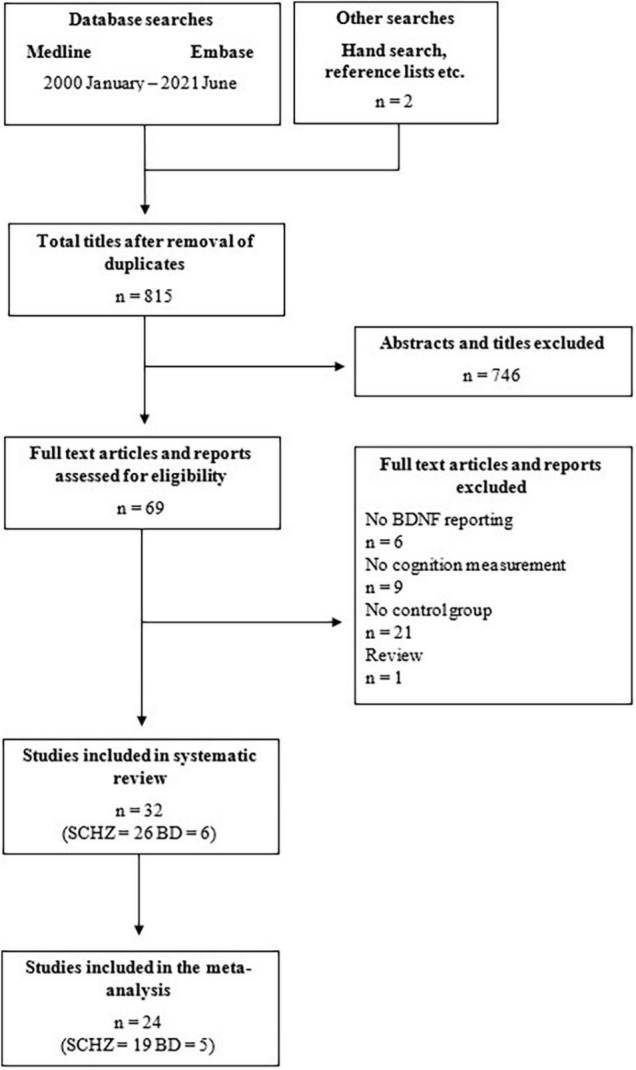
PRISMA flowchart demonstrating the search strategy that was utilized for the systematic review and meta-analysis.

A summary of article selection for the systematic review is presented in the PRISMA chart (Figure 1). A total of 815 articles were identified and 69 were determined as potentially eligible to be included in the review based on the titles and abstracts. After evaluating the articles fully, only 32 met the inclusion criteria as in several articles there were no control group present (21 studies) or the reporting on BDNF levels (6 studies) or cognitive functions (9 studies) was inadequate. The majority of studies (26 studies) were conducted with schizophrenia patients; 16 of them were with either first-episode patients (FEP) (34–41) or patients with chronic schizophrenia (CH) (42–48). In the bipolar studies there were patients in euthymic state (49–52) and in depressive (53) and manic (50) episodes involved. No studies involving schizoaffective patients were obtained which is a relevant shortcoming. Altogether, 4,754 schizophrenia and 476 bipolar disorder patients were compared to 3,526 healthy controls in the systematic review. In terms of level of evidence, most of the studies were level B according to the rating system by Siwek et al., meaning that the design of the studies were of lower quality, mostly case-control studies, and there were only a few randomized controlled trials (level A) (54).

Studies where BDNF levels or RBANS scores were not reported for the total patient population were excluded from the meta-analyses. The first analysis that compared baseline BDNF levels between schizophrenia patients and healthy controls included 19 studies. The second analysis that looked at baseline BDNF levels of bipolar disorder patients versus healthy controls included five studies (49, 51–53, 55). The third and final analysis examined the differences of baseline RBANS scores between schizophrenia patients and healthy controls also included 5 studies (34, 48, 56–58).

### Blood Brain-Derived Neurotrophic Factor Levels

Circulating BDNF concentrations were measured either in the plasma (9 studies) or in the serum (23 studies). Statistically significant difference between patients and healthy controls was detected in 25 studies (21 with schizophrenia and 4 with bipolar disorder patients) as summarized in Table 1. Importantly, 24 of these studies reported decreased blood BDNF levels in patients compared to controls; only one study by Asevedo et al. found higher BDNF levels in schizophrenia patients than in controls (59). Out of the seven studies where no significant difference was reported, two were conducted with euthymic bipolar disorder patients (49, 52), while the rest was with schizophrenia patients (37, 46, 60–62). The other two studies examining euthymic bipolar patients found reduced BDNF levels compared to healthy controls, nonetheless, it was highlighted that the levels were still higher than what was found in manic bipolar patients (50, 51).

**TABLE 1:** Summary of studies.

Study	Diagnosis	Patient N	Control N	BDNF type	BDNF in patients versus controls	BDNF ES (95% CI)	Cognition measures	Cognition in patients versus controls	BDNF-cognition relationship	Level of evidence (54)
Asevedo et al. (59)	SZ	30	27	Plasma	↑	1.04 (0.52; 1.57)	Verbal learning, verbal fluency, working memory, set shifting, inhibition, executive function tests	Deficits in verbal learning	BDNF levels positively correlated with semantic generation tasks	B

Carlino et al. (42)	CH-SZ	40	40	Serum	↓	–	Processing speed, attention, executive function, working memory tests	Significantly poorer neurocognitive performance	Serum truncated-BDNF abundance predicted for high cognitive deficits	B

Chang et al. (55)	BD-II	228	135	Plasma	↓	−0.23 (−0.44; −0.01)	WMS	Significantly lower scores on 5 subtests of WMS	BDNF more likely to be associated with clinical characteristics than with memory	B

Chou et al. (49)	EU-BD-I	23	33	Plasma	NS	−0.02 (−0.55; 0.51)	Attention, memory, executive function tests	Cognitive deficits present	Deficits in cognition not significantly correlated with BDNF except two items from tests	B

Dell’Osso et al. (53)	D-BD-I	16	15	Plasma	↓	−1.71 (−2.55; −0.87)	Cognitive disturbances factor score form HRSD	NA	BDNF levels may be related to severity of depression and retardation symptoms	B

Dias et al. (52)	EU-BD-I	65	50	Serum	NS	0.19 (−0.18; 0.56)	Attention and mental control, perceptual-motor skills, executive functions, verbal fluency and abstraction, visuospatial attention, memory tests	Significantly worse results on 11 out of the 16 neurocognitive tests	Significant positive association between serum BDNF levels and a test of verbal fluency in both BD patients and controls	B

Dong et al. (64)	SZ	818	467	Serum	↓	CLZ, MA: −0.85 (−1.02; −0.67) CLZ, FE: −0.13 (−0.40; 0.15) RISP, MA: −0.86 (−1.08; −0.64) RISP, FE: −0.55 (−0.86; −0.24) TYP, MA: −0.99 (−1.19; −0.78) TYP, FE: −0.09 (−0.47; 0−29)	RBANS	Significantly lower scores	Association between BDNF and cognitive performance in only male patients and female patients taking typical antipsychotics	B

Hori et al. (46)	CH-SZ	86	51	Serum	NS	−0.32 (−0.67; 0.03)	IGT	Significantly lower scores in IGT	Negative correlation between BDNF levels and mean net scores on the trials in the final two blocks	B

Hori et al. (60)	SZ	146	51	Serum	NS	−0.24 (−0.56; 0.08)	BACS	NA	Negative correlations between serum BDNF levels and scores for verbal memory, attention and processing speed	B

Li et al. (70)	SZ	472	225	Serum	↓	−1.56 (−1,72; −1.40)	RBANS	Significantly lower RBANS total score	Serum BDNF independently positively correlated with attention and immediate memory	B

Man et al. (34)	FEP-SZ	80	80	Serum	↓	−1.22 (−1.56; −0.88)	RBANS	Significantly lower cognitive performance on the RBANS total and four of its five subscale scores	No significant correlation between BDNF and any index or total scores of RBANS	B

Mora et al. (50)	EU-BD and MA-BD	84 (52 EU; 32 MA)	49	Serum	↓	EU: −0.50 (−0.89; −0.10) MA: −0.89 (−1.35; −0.44)	Executive function, selective attention, inhibition, processing speed, cognitive flexibility, sustained attention, perseverative behavior, verbal learning, recall, recognition, visual memory tests	Worse performance in executive functioning, inhibition, processing speed, verbal and visual memory	BDNF levels associated with executive functioning and verbal memory, together with other demographic variables	B

Niitsu et al. (61)	SZ	63	52	Serum	NS	0.17 (0,20; 0.54)	WAIS-R, VFT, WCST, TMT, Stroop test, DSDT	Significantly worse performance on all tests	Serum BDNF levels related to the impairment of verbal working memory in patients	B

Penadés et al. (62)	SZ	70	15	Serum	NS	0.25 (−0.31; 0.81)	Global cognition, working memory, processing speed, verbal memory, non-verbal memory, executive function tests	Significantly worse performance on most tests	No significant correlation between serum BDNF level and cognition	A

Qu et al. (35)	DN-FEP-SZ	256	177	Serum	↓	M: −1.07 (−1.28; −0.86) F: −0.85 (−1.09; −0.62)	RBANS	Cognitive function decreased	No association between BDNF and cognitive function	B

de Azua et al. (36)	FEP-SZ	45	45	Plasma	↓	−0.78 (−1.20; −0.35)	Learning ability, immediate and delayed memory, abstract thinking, and processing speed tests	Cognitive performance of patients significantly worse	Plasma BDNF levels at 6 months after first hospitalization positively associated with several cognitive domains	B

Rybakowski et al. (51)	EU-BD-I	60	60	Plasma	↓	−0.43 (−0.80; −0.07)	CANTAB	Lithium-treated patients had poorer results on all domains of neuropsychological tests	Performance on neuropsychological tests and plasma BDNF levels in excellent lithium responders is different compared to patients lacking the optimal effect of lithium but not different compared to matched healthy controls	B

Tang et al. (82)	SZ	109	40	Serum	↓	DS: −2.44 (−2.86; −2.02) NDS: −2.25 (−2.66; −1.84)	Processing speed, attention, executive function, working memory tests	Significantly worse performance	No correlations between BDNF levels and the cognitive tests in SZ and HC groups	B

Theleritis et al. (37)	FEP-SZ	87	152	Plasma	NS	0.26 (−0.01; 0−52)	IQ, verbal memory and learning, visual memory, executive function, working memory, attention, concentration, processing speed, verbal fluency tests	NA	No association between BDNF plasma levels and cognitive functions	B

Vinogradov et al. (43)	CH-SZ	56	15	Serum	↓	−0.59 (−1.16; −0.03)	MCCB	Decrement in cognitive functioning (∼ 1 SD below the normal mean)	No significant association between change in BDNF and change in global cognition	A

Wei et al. (44)	CH-SZ	189	60	Serum	↓	−1.00 (−1.30; −0.70)	Executive function test	Executive function impaired	BDNF may be a useful biomarker for executive dysfunction	B

Wu et al. (45)	CH-SZ	83	52	Serum	↓	TD: −1.00 (−1.43; −0.57) WTD: −0.65 (−1.05; −0.25)	RBANS	Significantly lower scores in almost all subscales	No significant associations between BDNF and RBANS total score or any cognitive index	B

Wu et al. (38)	DN-FEP-SZ	354	152	Serum	↓	−1.08 (−1.27; −0.89)	RBANS	Extensive cognitive impairment	No significant association between BDNF levels and RBANS total score or its index scores	B

Xiao et al. (63)	DN-FEP-SZ	58	55	Serum	↓	−0.99 (−1.39; −0.65)	Verbal fluency, attention and processing speed, attention distribution, working memory, motor speed, and executive function tests	Significantly worse on nearly all neurocognitive tests	BDNF levels positively correlated with the animal subscale of the VFT and negatively correlated with TMT-part B scores	B

Xiu et al. (39)	SZ	232	60	Serum	↓	−0.81 (−1.10; −0.53)	Executive function tests	Significantly lower scores	Lower BDNF levels were correlated with executive dysfunction	B

Xiu et al. (56)	DN-FEP-SZ	327	391	Serum	↓	−0.88 (−1.04; −0.73)	RBANS	Significantly lower scores	No relationship between BDNF and cognitive impairments	B

Yang et al. (40)	FEP-SZ, CH-SZ	65 34 FEP, 31 CH	35	Plasma	↓	FEP: −0.44 (−0.91; 0.04) CH: −0.62 (−1.11; −0.13)	MCCB	Index scores remarkably lower	Low BDNF levels were associated with cognitive impairments	B

Zhang et al. (69)	SZ	575	405	Serum	↓	Val/Val: −0.77 (−1.02; −0.52) Val/Met: −0.82 (−1.00; −0.65) Met/Met: −0.88 (−1.15; −0.60)	RBANS	Significantly lower in cognitive scores in nearly all subscales	Higher serum BDNF levels were associated with better cognitive function	B

Zhang et al. (48)	CH-SZ	251	206	Serum	↓	−0.93 (−1.13; −0.74)	RBANS	Significantly lower scores	BDNF positively associated with immediate memory	B

Zhang et al. (47)	CH-SZ	248	188	Serum	↓	−0.91 (−1.10; −0.72)	RBANS	Worse performance on most of the cognitive tasks	BDNF positively associated with immediate memory in female patients	B

Zhang et al. (57)	SZ	108	47	Serum	↓	−1.70 (−2,05; −1.36)	RBANS	Significantly lower scores	Metabolic adverse effects of olanzapine may aggravate cognitive dysfunction in patients with schizophrenia through an interaction between BDNF	B

Zhang et al. (58)	AC-SZ	68	47	Plasma	↓	−0.52 (−0.89; −0.14)	RBANS	Decreased compared to controls	Increase in plasma levels of BDNF significantly correlated with the change in the RBANS total scores	B

*AC, acute; BACS, Brief Assessment of Cognition in Schizophrenia; BD, bipolar disorder; BDNF, brain-derived neurotrophic factor; CANTAB, Cambridge Neuropsychological Test Automated Battery; CH, chronic; CLZ, clozapine; D, depressed; DN, drug-naïve; DS, deficit schizophrenia; DSDT, Digit Span Distraction Test; ES, effect size; EU, euthymic; F, female; FEP, first episode; HRSD, Hamilton Rating Scale for Depression; IGT, Iowa Gambling Task; MA, manic; M, male; MCCB, MATRICS Consensus Cognitive Battery; NDS, non-deficit schizophrenia; NA, not available, NS, not significant; RBANS, Repeatable Battery for the Assessment of Neuropsychological; RISP, risperidone; SZ, schizophrenia; TD, tardive dyskinesia; TMT, Trail Making Test; TYP, typical; VFT, Verbal Fluency Test; WAIS-R, Wechsler Adult Intelligence Scale Revised; WCST, Wisconsin Card Sorting Test; WMS, Wechsler Memory Scale; WTD, without tardive dyskinesia.*

When analyzing the effect sizes, large effect size (Hedges’ *g* of 0.8 or larger) was detected in 15 studies (1 study with bipolar and 14 with schizophrenia patients), medium (Hedges’ *g* of 0.5 to 0.8) in 5 studies, and small (Hedges’ *g* under 0.5) in 11 studies (Table 1). Large effect sizes were predominantly acquired in studies with first episode schizophrenia (34, 35, 38, 41, 63). In contrast, small effect sizes were more likely to be seen in studies involving bipolar patients (4 out of 6 studies) (49, 51, 52, 55). The smallest effect sizes were associated with patients with CH and/or patients receiving antipsychotic medication monotherapy (40, 46, 58, 60, 61). Importantly, female patients also seemed to have BDNF levels closer to normal compared to males (52, 64).

**FIGURE 2:**
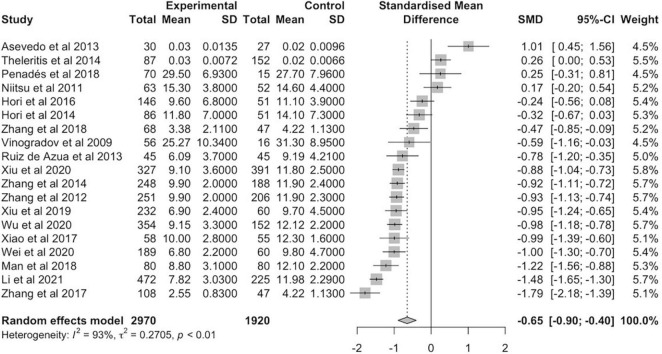
Forest plot of standardized mean difference (SMD) in BDNF levels found in blood of patients with schizophrenia and healthy controls.

**FIGURE 3:**
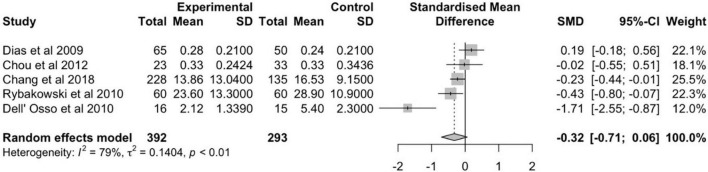
Forest plot of standardized mean difference (SMD) in BDNF levels found in blood of patients with bipolar disorder and healthy controls.

The meta-analysis of 19 schizophrenia studies was conducted with a total of 2,970 patients versus 1,920 healthy controls (Figure 2). The random effects estimate showed a moderate reduction of BDNF levels in schizophrenia patients compared to healthy controls (*g* = −0.65, 95% CI: −0.90 to −0.40). The level of heterogeneity was high (*I*^2^ = 93%, *p* < 0.01). The meta-analysis of the 5 bipolar disorder studies involved a total of 392 patients and 293 controls (Figure 3). In case of bipolar disorder patients, the random effects model reported a small reduction of BDNF levels in bipolar disorder patients in contrast to health controls (*g* = −0.32, 95% CI: −0.71 to 0.06) with slightly lower heterogeneity (*I*^2^ = 79%, *p* < 0.01).

### Cognitive Dysfunction

The assessment of cognitive functions varied within the reviewed literature. Most commonly (12 studies) the RBANS (65) was applied, whereas 7 studies used other validated scales such as the MATRICS™ Consensus Cognitive Battery (MCCB) (40, 43, 66), the Cambridge Neuropsychological Test Automated Battery (CANTAB) (51, 67) or the Brief Assessment of Cognition in Schizophrenia (BACS) (60, 68). The RBANS is a brief test that evaluates five indexes: immediate memory, visuospatial/constructional, language, attention, and delayed memory (65). In contrast, the BACS measures cognition functions *via* verbal memory, verbal fluency, working memory, motor speed, attention, and executive functioning (68), while the MCCB evaluates peed of processing, attention/vigilance, working memory, verbal learning, visual learning, reasoning and problem solving, and social cognition (66). The rest of the studies chose a combination of individual assessments that measured specific cognitive domains such as executive functioning, inhibition, or different aspects of memory (e.g., verbal or visual memory) *via* tests such as the Wisconsin Card Sorting Test (WCST) or specific parts of the Wechsler Adult Intelligence Scale (WAIS) (44, 50, 59).

Importantly, almost all studies (29 out of 32) reported significant difference between patients and controls, with patients exhibiting deficits in several cognitive domains. Concerning schizophrenia studies, differences between first episode and chronic patients were detected; higher scores were found in the chronic population compared to the first-episode population on the RBANS, with mean scores of 70.3 and 66.0, respectively (Table 2). Differences between males and females were also prevalent; female patients, especially in chronic populations, had lower cognitive impairment than male patients (35, 47, 64).

**TABLE 2:** Baseline RBANS scores in first episode and chronic schizophrenia patients.

Study	Schizophrenia patients		Healthy controls		Effect size	95% confidence interval
	Subject (sex)	Mean RBANS score (SD)	Subject (sex)	Mean RBANS score (SD)		
**First-episode schizophrenia**						
Man et al. (34)	80	64.0 (12.9)	80	79.0 (12.3)	−1.19	−1.50, −0.88
Qu et al. (35)	160 (M)	66.4 (14.5)	208	82.6 (13.1)	−1.16	−1.39, −0.94
	118 (F)	67.0 (17.6)	181	80.5 (15.3)	−0.82	−1.06, −0.58
Xiu et al. (56)	256	66.6 (15.9)	180	80.2 (15.0)	−0.88	−1.07, −0.69
Summary, means	685	66.0 (15.2)	860	81.6 (14.5)	−1.01	−1.25, −1.77
**Chronic schizophrenia**						
Dong et al. (64) (CLZ)	357 (M)	64.9 (14.7)	193 (M)	80.2 (15.0)	−1.02	−1.20, −0.83
	63 (F)	72.6 (17.4)	274 (F)	80.0 (15.3)	−0.46	−0.74, −0.19
Dong et al. (64) (RIS)	135 (M)	64.2 (16.0)	193 (M)	80.2 (15.0)	−1.02	−1−26, −0.79
	48 (F)	73.2 (14.3)	274 (F)	80.0 (15.3)	−0.44	−0.75, −0.13
Dong et al. (64) (TYP)	184 (M)	64.6 (13.9)	193 (M)	80.2 (15.0)	−1.06	−1.28, −0.85
	31 (F)	78.1 (18.4)	467 (F)	80.0 (15.3)	−0.12	−0.48, 0.24
Wu et al. (45) (WTD)	35	63.9 (9.1)	52	82.4 (12.5)	−0.74	−1.14, −0.33
	48	73.4 (11.6)	52	82.4 (12.5)	−1.62	−2.11, −1.13
Zhang et al. (48)	251	71.7 (16.4)	206	76.9 (16.0)	−0.33	−0.51, −0.14
Zhang et al. (47)	216 (M)	71.1 (15.2)	72 (M)	79.6 (13.1)	−0.57	−0.84, −0.30
	63 (F)	75.1 (17.1)	90 (F)	76.9 (14.8)	−0.11	−0.43, 0.21
Summary, means	1,431	78.1 (14.9)	2,066	79.9 (13.9)	−0.68	−1.25, −0.77

*CLZ, clozapine; F, female; M, male; RISP, risperidone; SD, standard deviation; TYP, typical; WTD, without tardive dyskinesia.*

**FIGURE 4:**
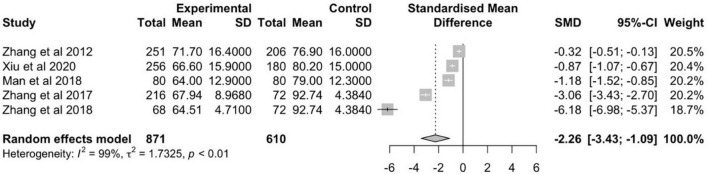
Forest plot of standardized mean difference (SMD) in total mean RBANS scores measured in patients with schizophrenia and healthy controls.

The meta-analysis of 5 schizophrenia studies was conducted with a total of 871 patients versus 610 healthy controls (Figure 4). The random effects estimate showed a large reduction of RBANS scores in schizophrenia patients compared to healthy controls (*g* = −2.26, 95% CI: −3.43 to −1.09). The level of heterogeneity was high (*I*^2^ = 99%, *p* < 0.01).

In case of bipolar disorder, patients in all states (manic, depressive or euthymic) were found to perform worse than controls on the different cognitive tests (49–52). Interestingly, significant difference between manic compared to euthymic patients was found in only one domain (verbal memory) in a study by Mora et al. (50). Nonetheless, the scores of euthymic patients were still lower than that of healthy controls (50). Similar results were acquired in patients treated with lithium, where poorer results on all cognitive domains compared to controls were reported (51). Importantly, the study also highlighted that excellent lithium responders had numerically better results than non-excellent responders (51). Due to heterogeneity of cognition measurements no meta-analysis could be conducted in patients with bipolar disorder.

### Correlation Between Blood Brain-Derived Neurotrophic Factor Levels and Cognitive Dysfunction

Correlations between circulating BDNF levels and cognitive functions in patients were calculated in 26 studies, out of which 16 studies reported Pearson’s correlation coefficients, 5 Spearman’s correlation coefficients and 5 partial correlation coefficients. Again, most of the studies correlated cognitive functions to BDNF serum levels, and only a few to plasma levels. In terms of cognitive functions, total scores, index scores, or individual test scores were included in the correlation analyses. All in all, 19 studies found significant correlations between circulating BDNF levels; correlation coefficients and *p*-values are shown in Table 3.

**TABLE 3:** Correlations between BDNF levels and cognition.

Study	Diagnosis	BDNF type	Cognition measurement	Correlation measure	Correlation coefficient
Asevedo et al. (59)	SZ	Serum	Semantic generation task	Spearman’s correlation	0.38*
			Letter memory task		−0.45*

Carlino et al. (42)	CH-SZ	Serum	TMT Part B	Partial correlation	0.55*** (low truncated BDNF)
			Digit symbol coding		0.36* (low truncated BDNF)
			Digit span forward		0.36* (low truncated BDNF)

Chou et al. (49)	EU-BD	Plasma	Sounds RT (divided attention)	Partial correlation	Data missing*
			Faces 2 true positive (faces memory)		Data missing**

Dias et al. (52)	EU-BD	Serum	Test of verbal fluency (COWAT)	Pearson’s correlation	0.26*

Dong et al. (64)	SZ	Serum	RBANS total score	Pearson’s correlation	0.18** (CLZ, M)
					0.39** (RISP, M)
					0.30** (TYP, M)
					0.55* (TYP, F)

Hori et al. (46)	CH-SZ	Serum	Card block 61–80 (IGT)	Pearson’s correlation	0.23*
			Card block 81–100 (IGT)		0.27*

Hori et al. (60)	SZ	Serum	Verbal memory (BACS)	Pearson’s and Spearman’s correlation	0.19*
			Attention and processing speed (BACS)		0.16*

Li et al. (70)	SZ	Serum	RBANS total score	Pearson’s correlation	0.33*** (without T2DM)

Niitsu et al. (61)	SZ	Serum	Information subscale (WAIS-R)	Spearman’s correlation	0.29*

Wei et al. (44)	CH-SZ	Serum	VFT total score	Partial correlation	0.33*

Wu et al. (45)	CH-SZ	Serum	RBANS total score	Pearson’s correlation	−0.38* (TD)
			Immediate memory index (RBANS)		0.32* (WTD) −0.36* (TD)
			Delayed memory index (RBANS)		−0.38* (TD)

Wu et al. (38)	DN-FEP-SZ	Serum	Delayed memory index (RBANS)	Pearson’s correlation	−0.26* (high−BDNF)

Xiao et al. (41)	DN-FEP-SZ	Serum	TMT Part B	Spearman’s correlation	−0.40**
			VFT-animals		0.27*

Xiu et al. (39)	SZ	Serum	VFT total score	Partial correlation	0.30*
			WCST sub-score		−0.27*

Yang et al. (40)	SZ	Plasma	Learning and memory (MCCB)	Pearson’s correlation	0.28*

Zhang et al. (69)	SZ	Serum	RBANS total score	Pearson’s correlation	0.21*

Zhang et al. (48)	CH-SZ	Serum	Immediate memory index (RBANS)	Pearson’s correlation	0.23***

Zhang et al. (47)	CH-SZ	Serum	RBANS total score	Pearson’s correlation	0.34** (F)
			Immediate memory index (RBANS)		0.51*** (F)

Zhang et al. (58)	AC-SZ	Plasma	RBANS total score	Spearman’s correlation	0.28*
			Attention index (RBANS)		0.27*

p-Value: *<0.05, **<0.01, ***<0.001.

*AC, acute; BACS, Brief Assessment of Cognition in Schizophrenia; BD, bipolar disorder; BDNF, brain-derived neurotrophic factor; CH, chronic; CLZ, clozapine; COWAT, Controlled Oral Word Association Test; DN, drug naïve; F, female; FEP, first episode; EU, euthymic; IGT, Iowa Gambling Task; M, male; MCCB, MATRICS Consensus Cognitive Battery; RBANS, Repeatable Battery for the Assessment of Neuropsychological Status; RISP, risperidone; SZ, schizophrenia; TD, tardive dyskinesia; TMT, Trail Making Test; TYP, typical; VFT, Verbal Fluency Test; WAIS-R, Wechsler Adult Intelligence Scale Revised; WCST, Wisconsin Card Sorting Test; WTD, without tardive dyskinesia.*

Most studies reported negligible (*r* < 0.3) (38–40, 46, 48, 52, 58, 60, 61, 63, 64, 69) or low (0.3 < *r* < 0.49) (39–42, 44, 45, 59, 64, 70) positive correlations between circulating BDNF levels and cognitive functions and only three studies found moderate (0.5 < *r* < 0.7) correlations (42, 47, 64). For instance, Dong et al. reported moderate positive correlation between baseline serum BDNF level and RBANS total score but only in female patients taking typical antipsychotic medications (*r* = 0.55, *p* < 0.05) (64). In contrast, correlations between BDNF serum levels and RBANS total scores were low in male patients taking typical antipsychotic medications (*r* = 0.30; *p* < 0.01) or risperidone (*r* = 0.39; *p* < 0.01) (64). Similarly, Zhang et al. found moderate positive correlation between BDNF serum levels and immediate memory index score from RBANS but only in chronic female schizophrenia patients (47). Finally, Carlino et al. found moderate positive correlation between low truncated-BDNF expression and performance on Trail Making Test Part B (*r* = 0.55; *p* < 0.001) in CH patients (42).

In general, significant correlations between circulating BDNF levels and cognitive assessments were more prevalent in the CH population, with 6 out of 7 studies reporting statistically significant correlation coefficients (42, 44–48). The specific domains associated with circulating BDNF levels were immediate (45, 47, 69), delayed (45) and working memory (42), decision making (46), speed of processing (42), executive (42), and verbal functioning (44). Interestingly, Wu et al. found different correlation directions depending on whether patients had tardive dyskinesia or not; serum BDNF levels of patients with TD correlated negatively with RBANS total score, and immediate and delayed memory indexes (45).

In case of (drug naïve) first episode patients, non-significant correlations between circulating BDNF levels and cognitive functioning were detected in the majority of studies (34, 35, 56). Only two studies by Wu et al. and Xiao et al. found significant correlations; Wu et al. reported negative correlation between delayed memory index from RBANs and serum BDNF levels (*r* = −0.26; *p* < 0.05), however only in patients with high baseline BDNF levels (38), Xiao et al. found negative correlation between serum BDNF levels and executive functioning (*r* = −0.40; *p* < 0.01) and positive correlation with verbal function (*r* = 0.27; *p* < 0.05) (41).

The rest of the studies examining schizophrenia detected significant correlations between circulating BDNF levels and semantic generation task (59), and verbal memory, attention and processing speed (60), verbal and executive functioning (39), and RBANS total score (58, 69, 70). Interestingly, correlations were different for schizophrenia patients with and without type 2 diabetes mellitus in a study by Li et al.; serum BDNF levels correlated with total RBANS score in non-diabetic schizophrenia patients only (*r* = 0.33; *p* < 0.001) (70).

Finally, in bipolar patients, circulating BDNF levels were reported to significantly correlate with specific domains of cognition in 2 out of the 3 studies that calculated coefficients, namely divided attention (*p* < 0.05) (49), faces memory (*p* < 0.01) (49), and verbal fluency (*r* = 0.26; *p* < 0.05) (52).

### Changes in Brain-Derived Neurotrophic Factor Levels and Cognitive Dysfunction After Treatment

Altogether, 4 of the 32 reviewed studies examined the changes in BDNF levels and cognitive functions before and after treatment, all with schizophrenia patients. Two studies focused on the effects of pharmacotherapy (38, 58) and two studies on the impact of non-pharmacological interventions such as cognitive remediation (62) and computerized auditory training (43) as summarized in Table 4. In case of the latter, 56 schizophrenia outpatients were randomized to 10 weeks of computerized auditory training or control condition (computer game) and were compared to 16 matched healthy controls. According to the results, statistically significant change was detected in global cognition as well as BDNF levels in response to the training (43). By week 10, the BDNF levels in patients were comparable to that of healthy controls; at baseline these were significantly lower than of the healthy controls (43). Nonetheless, no significant association was found between the changes in BDNF levels and the cognitive measurements (43). The other study that examined the effects of non-pharmacological treatment on BDNF levels and cognitive impairment randomized 70 patients to either cognitive remediation therapy (CRT) or social skills training (control group) for 4 weeks (62). Although some improvements in cognition were detected, the authors could not report any significant changes in serum BDNF levels in response to the CRT (62). Moreover, the association between cognitive improvements and BDNF levels were also non-significant (62). Both RCTs evaluated cognition *via* the MCCB (43, 62).

**TABLE 4:** Brain-derived neurotrophic factor levels and cognition scores at baseline and after treatment.

Study	Patient (*N*)	Treatment	BDNF, mean (SD)		Cognition measure	Cognition, mean (SD)	
			Before	After		Before	After
Penadés et al. (62)	35	4-month cognitive remediation	26.1 (7.4)	27.9 (9.1)	Global cognition	43.26 (4.62)	48.48 (4.32)
					Working memory	47.95 (9.91)	50.35 (8.84)
					Processing speed	43.15 (7.21)	48.82 (6.28)
					Verbal memory	37.99 (7.86)	44.75 (7.71)
					Non-verbal memory	44.24 (8.35)	47.90 (6.12)
					Executive function	40.88 (7.94)	49.11 (6.55)
					Quality of life EQ-5D	4.79 (1.12)	6.74 (1.12)

Vinogradov et al. (43)	29	50-h computerized auditory training	25.3 (10.3)	32.2 (15.1)	Global cognition	–	–
					Speed of processing		
					Verbal working memory		
					Verbal learning		
					Verbal memory		
					Problem solving		
					Non-verbal working memory		
					Visual learning		
					Visual memory		
					Social cognition		

Wu et al. (38)	190	12-week risperidone monotherapy	9.1 (3.3)	10.8 (6.3)	RBANS	–	–

Zhang et al. (58)	68	12-week olanzapine monotherapy	3.4 (2.1)	4.7 (1.7)	RBANS	322.57 (23.55)	339.34 (43.51)

*N, number; RBANS, Repeatable Battery for the Assessment of Neuropsychological Status; SD, standard deviation.*

In terms of pharmacological therapies, Zhang et al. designed a 12-week open-label, prospective observational trial to examine the effects of olanzapine on BDNF and cognitive functioning and thus to evaluate if BDNF can act as a biomarker for cognition (58). At baseline, the 95 patients exhibited significantly worse cognitive performance as measured by RBANS and lower plasma BDNF levels than the 72 controls (58). In response to olanzapine treatment, significant improvements in immediate memory, attention, and total RBANS score as well as increased plasma BDNF levels compared to baseline were detected (58). Importantly, the increase in BDNF plasma levels showed correlation with the change in the RBANS scores (58). Based on the results, the authors concluded that plasma BDNF levels might be a potential biomarker for cognitive functioning in patients with acute schizophrenia (58).

Similarly, Wu et al. conducted a 12-week, flexible-dose, prospective, observational trial in 354 drug-naïve FEP with schizophrenia (38). The aim was to evaluate the impact of risperidone treatment on serum BDNF levels and cognitive functioning measured by RBANS (38). According to the results, poorer cognitive functioning and lower serum BDNF levels were detected at baseline in patients compared to 152 controls (38). In response to treatment, significant improvement in memory, delayed memory and RBANS total score as well as slight increase in BDNF levels was found (38). Interestingly, when separating patients to low-BDNF and high-BDNF baseline groups, different responses to antipsychotic medication were acquired (38). Those in the low-BDNF group had increased, while those in the high-BDNF group had decreased plasma levels after risperidone treatment (38). In addition, correlations between lower BDNF levels and delayed memory were also detected, but only in patients who had higher baseline BDNF levels (38).

## Discussion

To our knowledge this is the first systematic review and meta-analysis that examined circulating BDNF levels and cognitive dysfunction in patients on the schizophrenia-bipolar spectrum. The aim of the paper was threefold: to update the existing literature regarding the differences between patients and healthy controls in blood BDNF levels and cognitive functioning, to compare patients with schizophrenia, bipolar disorder, and schizoaffective disorder in terms of circulating BDNF levels and cognitive dysfunction, and to understand the relationship between BDNF and cognition in these patient populations. The relevance of the results is discussed below.

The results confirmed that there is a moderate reduction in patients with schizophrenia and small reduction in patients with bipolar disorder in serum or plasma BDNF levels compared to healthy controls. The results are in line with previous meta-analyses that also found moderate quality evidence of reduced blood BDNF levels in these patient groups (16, 71–73). Similarly to the results of a comparative meta-analysis by Fernandes et al., the present study also agrees that the decrease in circulating BDNF levels compared to healthy controls is greater in acute patients than in those in chronic or euthymic states (73). Differences in blood BDNF levels also seem to depend on several other factors including sex, age, or medication, which was again shown by previous research as well (74, 75).

In contrast to circulating BDNF levels, cognitive impairment was found to be pronounced in all states and stages of the disorders, confirming that indeed cognitive deficits are a core feature of the schizophrenia-bipolar spectrum. Interestingly however, better scores on different cognition assessments were reported in patients with CH compared to patients in first episode. In terms of the relationship between circulating BDNF levels and cognitive functioning, significant but negligible correlations were found in more than one third of the reviewed studies. Differences between patient groups were also prevalent in this aspect of the analysis as well; significant correlations were more likely to be found in chronic patients compared to first episode patients, and in female patients compared to males.

All in all, circulating BDNF levels alone do not seem to be a valid biomarker of cognitive dysfunction in patients on the schizophrenia-bipolar spectrum. Although BDNF has been repeatedly found to be reduced in patients compared to healthy controls, the correlations between BDNF and cognition are weak. This is especially true for drug naïve first episode patients who have high levels of cognitive dysfunction and low levels of blood BDNF, yet the two are not correlated. Indeed, the relationship between cognition and BDNF is more pronounced in patients with CH, suggesting that factors such as age or state of disorder might be mediating this relationship. This has been proposed by previous reviews as well; although the meta-analysis by Bora et al. found correlation between cognitive symptoms and BDNF levels, they also concluded that the relationship between the two might be rather indirect (30). In addition, Fernandes et al. came to similar conclusions too, suggesting that reduced BDNF levels might be connected to the suppressive effects of stress (73).

If putting these results into context, it is likely that the reason why most reduction in BDNF levels was detected in first-episode, drug naïve patients is due to that fact that these patients experience the highest levels of stress. As the stress levels are lower in chronic, medicated, and euthymic patients, the BDNF levels are less influenced by it and hence correlations between cognitive symptoms are more prevalent. The fact that most correlations in this patient population were found in executive functioning, immediate memory, and processing speed – neurocognitive functions all mediated by the hippocampus and prefrontal cortex – further supports this notion.

Finally, it deserves attention from clinical point of view that blood BDNF levels and cognitive symptoms were found to improve after certain therapies and antipsychotic medications. In case of pharmacotherapy, the improvement in cognitive functioning and circulating BDNF levels were even correlated. Some experimental results suggest that D_3_ receptors may also play a role in influencing BDNF levels and cognitive improvement (76–79). Thus, further research needs to investigate whether novel medications targeting D_3_ receptors have different effects on BDNF levels in patients on the schizophrenia-bipolar spectrum and how these potential changes in BDNF levels would relate to cognitive impairment.

### Limitations

The main limitation of this systematic review is the heterogeneity of the studies; large differences in sample sizes, patient populations, BDNF measurements (plasma or serum) and cognitive scales were prevalent. Due to this heterogeneity the relationship between BDNF levels and cognitive dysfunction could not be quantified, as neither the Hedges–Olkin nor the Schmidt–Hunter method is suitable for a small number of heterogeneous studies (80). In addition, according to Rosenfeld et al., there is significant difference between BDNF serum and plasma levels, nonetheless as previous reviews, this review also analyzed serum and plasma BDNF levels together (81). Furthermore, in some articles, potential overlap in samples were detected, hence, introducing bias to the analysis. The systematic review also did not account for neither the maturity of BDNF nor BDNF polymorphism, which could play an important role in determining BDNF levels in the periphery. Finally, no studies with exclusively schizoaffective patients were obtained, hence, this patient group is missing from the schizophrenia-bipolar spectrum.

## Conclusion

While it has been confirmed that blood BDNF levels, especially during the acute phases are decreased, there are several factors that influence circulating BDNF levels making it unreliable as a biomarker of cognitive dysfunction alone. In contrast, circulating BDNF might be considered as a psychiatric state marker and thus, changes in BDNF levels in the plasma/serum should be evaluated in the context of a wider pattern of risk and protective factors such as inflammatory, immune, and metabolic parameters. Nonetheless, this does not necessarily mean that targeting BDNF would not influence cognition positively. Future research should investigate how different treatments influence circulating BDNF levels and cognitive symptoms, especially executive functioning, and memory, and whether there is a correlation between the changes detected.

## Data Availability Statement

The raw data supporting the conclusions of this article will be made available by the authors, without undue reservation.

## Author Contributions

ZD, IS, and PB contributed to conception of the manuscript. ZD wrote the first draft of the manuscript. All authors contributed to manuscript revision, read, and approved the submitted version.

## Conflict of Interest

ZD was an employee of Gedeon Richter Plc. The remaining authors declare that the research was conducted in the absence of any commercial or financial relationships that could be construed as a potential conflict of interest.

## Publisher’s Note

All claims expressed in this article are solely those of the authors and do not necessarily represent those of their affiliated organizations, or those of the publisher, the editors and the reviewers. Any product that may be evaluated in this article, or claim that may be made by its manufacturer, is not guaranteed or endorsed by the publisher.
